# Directional RNA deep sequencing sheds new light on the transcriptional response of *Anabaena *sp. strain PCC 7120 to combined-nitrogen deprivation

**DOI:** 10.1186/1471-2164-12-332

**Published:** 2011-06-28

**Authors:** Britt L Flaherty, F Van Nieuwerburgh, Steven R Head, James W Golden

**Affiliations:** 1 Division of Biological Sciences, University of California San Diego, La Jolla, CA 92093-0116, USA; 2 Laboratory of Pharmaceutical Biotechnology, Ghent University, Harelbekestraat 72, 9000 Ghent, Belgium; 3 Genomics Core Facility, The Scripps Research Institute, La Jolla, CA 92037, USA

## Abstract

**Background:**

Cyanobacteria are potential sources of renewable chemicals and biofuels and serve as model organisms for bacterial photosynthesis, nitrogen fixation, and responses to environmental changes. *Anabaena *(*Nostoc*) sp. strain PCC 7120 (hereafter *Anabaena*) is a multicellular filamentous cyanobacterium that can "fix" atmospheric nitrogen into ammonia when grown in the absence of a source of combined nitrogen. Because the nitrogenase enzyme is oxygen sensitive, *Anabaena *forms specialized cells called heterocysts that create a microoxic environment for nitrogen fixation. We have employed directional RNA-seq to map the *Anabaena *transcriptome during vegetative cell growth and in response to combined-nitrogen deprivation, which induces filaments to undergo heterocyst development. Our data provide an unprecedented view of transcriptional changes in *Anabaena *filaments during the induction of heterocyst development and transition to diazotrophic growth.

**Results:**

Using the Illumina short read platform and a directional RNA-seq protocol, we obtained deep sequencing data for RNA extracted from filaments at 0, 6, 12, and 21 hours after the removal of combined nitrogen. The RNA-seq data provided information on transcript abundance and boundaries for the entire transcriptome. From these data, we detected novel antisense transcripts within the UTRs (untranslated regions) and coding regions of key genes involved in heterocyst development, suggesting that antisense RNAs may be important regulators of the nitrogen response. In addition, many 5' UTRs were longer than anticipated, sometimes extending into upstream open reading frames (ORFs), and operons often showed complex structure and regulation. Finally, many genes that had not been previously identified as being involved in heterocyst development showed regulation, providing new candidates for future studies in this model organism.

**Conclusions:**

Directional RNA-seq data were obtained that provide comprehensive mapping of transcript boundaries and abundance for all transcribed RNAs in *Anabaena *filaments during the response to nitrogen deprivation. We have identified genes and noncoding RNAs that are transcriptionally regulated during heterocyst development. These data provide detailed information on the *Anabaena *transcriptome as filaments undergo heterocyst development and begin nitrogen fixation.

## Background

Cyanobacteria are photosynthetic prokaryotes that have evolved a wide array of metabolic capabilities [1]. Because of their high photosynthetic efficiency, variety of metabolic pathways, and genetic manipulability, they are a potential source of "green" chemicals and fuels [2,3]. Some cyanobacteria reduce atmospheric nitrogen to ammonia to support growth in nitrogen-deficient conditions [4]. Because nitrogen is often a limiting resource for growth, this gives nitrogen-fixing strains a competitive edge in some environments. Understanding the response to nitrogen deprivation, nitrogen fixation, and diazotrophic growth in cyanobacteria will shed light on basic mechanisms of bacterial genetic regulation and physiology. In addition, it may help to develop better strains of cyanobacteria for the production of renewable chemicals and biofuels.

The cyanobacterium *Anabaena *(*Nostoc*) sp. strain PCC 7120 grows as long filaments of photosynthetic vegetative cells in the presence of combined nitrogen. In an environment lacking combined nitrogen, about 7 to 10% of the cells terminally differentiate into nitrogen-fixing heterocysts. Heterocysts provide a microoxic environment for the expression of the oxygen-sensitive nitrogenase enzyme [5,6]. Single heterocysts are spaced about every 10-15 cells along filaments and they supply fixed nitrogen, probably in the form of amino acids, to neighboring vegetative cells [5]. Vegetative cells provide heterocysts with products of carbon fixation, probably as sucrose [7,8], thus creating a multicellular organism with two mutually dependent cell types. Heterocyst development involves the response of vegetative cells to nitrogen deprivation, the formation and maintenance of the pattern of the two cell types, differentiation of heterocysts from vegetative cells, and the adaptations made by vegetative cells to adjust to diazotrophic growth.

The differentiation of a vegetative cell into a heterocyst involves substantial changes in cell morphology and physiology [5,6]. Heterocysts deposit glycolipid and polysaccharide layers outside of their cell wall to limit the entry of atmospheric oxygen [9-11]. They lack photosystem II activity, which normally produces O_2_, and increase respiration to consume O_2 _that enters the cell. Heterocyst differentiation requires dramatic changes in transcription and some of the key components of this regulation are known. Nitrogen limitation is sensed by accumulation of 2-oxoglutarate (2-OG), the backbone for nitrogen assimilation. 2-OG enhances the DNA-binding activity of the transcription factor *ntcA *[12], which regulates expression of the response regulator *nrrA*, which is partially responsible for upregulation of *hetR *[13,14]. HetR, deemed the master regulator of heterocyst development, regulates the expression of many genes, including the glycolipid genes (*hgl*), exopolysaccharide genes (*hep*), and the *patS *gene, which encodes a peptide involved in heterocyst pattern formation [15].

Factors other than those described above are known to be involved in heterocyst development and have been identified through microarrays and genetic screens [16-22]. While these methods are powerful, microarrays and screens often overlook unannotated regions of the genome and antisense or noncoding transcripts. In addition, they lack sensitivity and do not provide information on UTR length or operon structure. Therefore, we have employed directional RNA-seq to analyze the transcriptome of *Anabaena *filaments during nitrogen step-down to identify and map all transcripts during heterocyst development [23-27].

Our RNA-seq data provide information on the UTR lengths of each mRNA transcript, on the transcription of sense and antisense or noncoding RNAs, and on the changes in expression of all transcripts whether or not they carry an annotation. The data show long 5' UTRs for many genes, likely with multiple transcriptional start or processing sites. In addition, our study identifies antisense transcription in the coding region or UTR of many genes known to be involved in heterocyst development. Finally, we detected new genes that are significantly upregulated in response to nitrogen deprivation.

## Results and Discussion

### Analysis of the transcriptional response to nitrogen step-down

We obtained RNA-seq data for total RNA isolated from *Anabaena *filaments grown with ammonium as a nitrogen source and at three times after nitrogen step-down from ammonium to dinitrogen in air. The nitrogen step-down produces relatively synchronous induction of heterocyst development. RNA-seq data were acquired from filaments at 0, 6, 12, and 21 hours after nitrogen step-down, which provides detailed transcriptome data at important stages of heterocyst development (GEO accession #GSE26633). At 0 hours, all cells in the filaments are actively growing photosynthetic vegetative cells. At 6 hours, the cells have responded to the nitrogen step-down and are expressing early heterocyst differentiation genes such as *hetR*, the master regulator of heterocyst development, and *patS*, which is involved in pattern formation. By 12 hours, proheterocysts are committed to complete differentiation and are expressing genes required for altering their morphology and physiology to become microoxic. By 21 hours, nearly all heterocysts appear fully formed, contain polar cyanophycin granules, and are actively fixing nitrogen.

RNA-seq expression data are presented as RPKM, or **r**eads **p**er **k**ilobase (kb) of CDS (coding sequence) model per **m**illion mapped reads in the sample [28], with the CDS model defined as the CDS plus 100 bp of 5' UTR. RPKM values and changes in RPKM value for the chromosome and six plasmids are presented as additional files 1, 2, 3, 4, 5, 6, and 7. These data can be examined and filtered in many ways. For example, for genes on the chromosome (additional file 1: Chromosome.xlsx) with a RPKM value of at least 2 (which includes only those genes with good read coverage) and a fold change of at least 5, there are 22 genes with increased expression by 6 hours after nitrogen step-down, 434 genes upregulated by 12 hours (including many known heterocyst morphogenesis genes), and 396 genes upregulated by 21 hours (including the nitrogen fixation genes). For genes on the chromosome with decreased expression, there are 6 genes downregulated at 6 hours after nitrogen step-down, 32 genes downregulated at 12 hours, and 35 genes downregulated at 21 hours.

Upregulation of nitrogen-fixation genes is the culminating event of heterocyst differentiation. The RNA-seq data provide detailed information on the expression of the known nitrogen-fixation genes as well as hypothetical and unknown genes that show the same pattern of regulation. For example, the data show very low levels of reads for *nifHDK *and other *nif *operons at 0, 6, and 12 hours after nitrogen step-down (when heterocysts are not yet fully formed). The reads for all *nif *operons and especially for the *nifHDK *genes are dramatically increased in the 21 h sample, when most heterocysts are fully differentiated (Table 1 and additional file 1: Chromosome.xlsx).

**Table 1:** Temporal response of the *nifHDK *operon and selected heterocyst glycolipid (*hgl*) and polysaccharide (*hep*) genes to nitrogen step-down

		RPKM			
		
Gene Name	Locus Name	0 h	6 h	12 h	21 h
*nifH*	all1455	0.89	0.77	6.51	1940.13
*nifD*	all1454	0.00	0.04	0.42	108.34
*nifK*	all1440	0.11	0.00	0.89	242.20
*hglC*	alr5355	0.12	0.26	0.39	5.83
*hglD*	alr5354	0.24	0.22	0.08	7.09
*hglE*	alr5351	0.04	0.05	0.63	21.05
*hepA*	alr2835	0.00	0.03	18.84	5.85
*hepB*	alr3698	0.00	0.15	6.84	2.78
*hepK*	all4496	5.27	5.68	12.80	10.79

Formation of the heterocyst envelope involves deposition of a polysaccharide outer layer followed by deposition of an inner glycolipid layer [9,11]. The RNA-seq data show that the genes responsible for heterocyst exopolysaccharide synthesis (*hep *genes) were upregulated by 12 hours after nitrogen step-down (Table 1). However, strong upregulation of the genes required for heterocyst glycolipid synthesis (*hgl *genes) did not occur until the 21-hour sample. These data show that during heterocyst morphogenesis, the polysaccharide genes are expressed first, likely depositing the stabilizing exopolysaccharide later; subsequently, *hgl *genes are expressed to produce the underlying glycolipid envelope layer, which together are required to help create a microoxic environment within the heterocyst.

**Figure 1:**
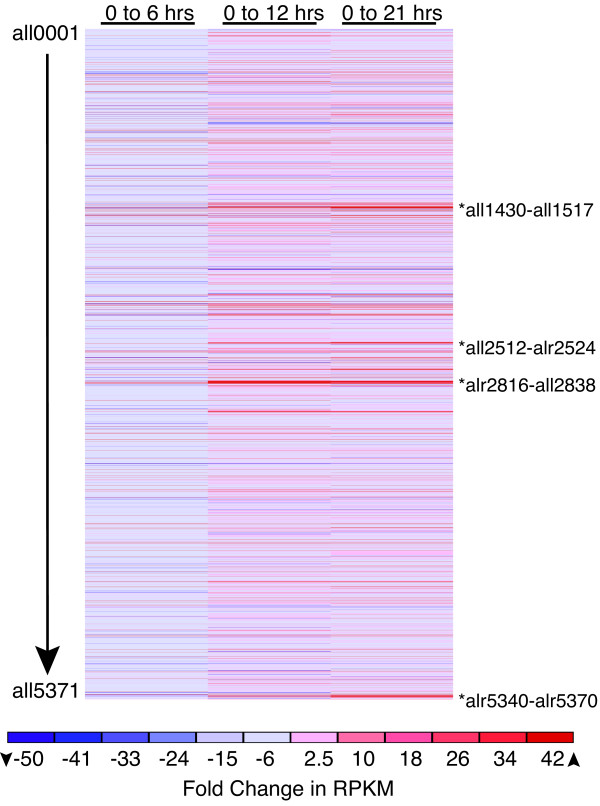
**Gene clusters upregulated during nitrogen deprivation**. The fold change in RPKM from 0 to 6, 12, and 21 hours after removal of combined nitrogen is represented in a heat map across the chromosome. Genes are shown in order from all0001 at the top to all5371 at the bottom. Clusters of genes that are upregulated and discussed in the text are marked with an asterisk and locus identifiers. The heat map was produced with The Broad Institute's GenePattern software and the dataset (additional file 8: RPKM GenePattern.gct) can be analyzed with the free GenePattern software [48].

In addition to identifying single genes that respond to nitrogen deprivation, we mapped gene clusters that were upregulated in response to nitrogen step-down. As expected, the region containing the major *nif *operons from *fdxH *(all1430)-*nifB *(all1517) (with the exception of the *nifD *and *fdxN *elements present in the vegetative cell chromosome [6]) was strongly upregulated by 21 hours in response to nitrogen deprivation (Figure 1 and additional file 8: RPKM GenePattern.gct). Another cluster of genes in the *patB *(all2512)-alr2524 region, which contains the cytochrome oxidase genes *coxBAC*, and many genes annotated as unknown or hypothetical, was strongly upregulated by 21 hours after nitrogen step-down. Genes in the region alr2816-all2838, which contains *hetC*, *hetP*, and *hepA*, as well as many genes annotated as encoding hypothetical, glycosyltransferases, and metabolic proteins, were upregulated by 12 hours after nitrogen step down. Finally, genes in the region alr5340-alr5370, which contains several *hgl *(heterocyst glycolipid) genes along with a number of hypothetical genes, were upregulated by 21 hours after nitrogen step-down. Each of these regions contains a number of genes and operons that are important for heterocyst development and nitrogen fixation.

### Identification of unstudied genes regulated in response to nitrogen deprivation

The RNA-seq data showed regulation of numerous genes in response to nitrogen step-down; including many that had not been previously identified as nitrogen-responsive in microarrays or genetic experiments. These genes are new candidates for the study of *Anabaena *heterocyst differentiation. We identified several new genes transcribed in response to nitrogen deprivation that have GO terms associated with regulation, including transcriptional regulators, two-component regulators, and kinases (Table 2 and additional file 1: Chromosome.xlsx). These genes may be involved in the regulatory pathways and transcriptional changes responsible for coordinating the expression of proteins required for heterocyst morphogenesis and nitrogen fixation.

**Table 2:** Unstudied regulatory genes with 5-fold or greater increase in expression by 6, 12, or 21 hours after nitrogen step down^1^

		RPKM fold change		
		
Gene name	Gene description	6 h	12 h	21 h
*typA *(all4140)^2^	GTP-binding protein TypA/BipA	-1.2	6.8	5.9
all0723^2^	probable GTP-binding protein	1.1	6.1	6.2
all2564^3^	pyruvate kinase	2.3	50.5	25.2
all4008^3^	pyruvate kinase	1.0	4.3	5.3
all0192^2^	serine/threonine kinase	6.5	45.4	52.8
alr1336^3^	serine/threonine kinase	1.9	3.2	6.3
all4838^3^	serine/threonine kinase	1.3	6.6	3.8
alr1044^2^	transcriptional regulator	1.5	6.1	2.1
all2237^2^	transcriptional regulator	1.1	5.5	4.6
alr2479^3^	transcriptional regulator	-1.2	7.9	2.8
alr2769^2^	transcriptional regulator	-2.5	3.0	8.3
alr3646^3^	transcriptional regulator	1.1	9.0	2.1
all3728^2^	transcriptional regulator	2.4	7.2	6.7
alr4564^2^	transcriptional regulator	1.6	3.9	5.3
alr0546^2^	two-component sensor histidine kinase	1.0	5.3	2.6
all3359^2^	two-component sensor histidine kinase	-1.3	5.7	8.4
alr4878^2^	two-component hybrid sensor and regulator	1.4	6.2	3.8
alr5188^3^	two-component response regulator	1.1	5.5	2.8

^1^Only unstudied genes with at least one-fold read coverage and annotated with the GO term "regulatory function" are presented.

^2^Genes that were not identified as being significantly upregulated in previous microarray analyses [13,49].

^3^Genes that were previously identified as being regulated in microarray data but that show a greater degree of upregulation in our RNA-seq data [13,49].

We also identified many transposase genes that are upregulated by 6 hours after nitrogen step-down (Table 3 and additional file 1: Chromosome.xlsx). Transposases are highly similar within a family and RNA-seq reads cannot always assign a sequence to a particular transposase locus for transposases within the same family. However, we can see that distinct families of transposons are upregulated in response to nitrogen step-down. Table 3 shows the average fold change in RPKM during nitrogen step-down for four families of transposons [29] that are upregulated at least 2-fold by 6 hours after nitrogen deprivation. It seems likely that transposase genes are turned on as a stress response to nitrogen deprivation.

**Table 3:** Transposase gene families with a 2-fold or greater increase in expression by 6 hours after nitrogen step-down1

	Average Fold Change in RPKM		
	
Transposon Family	0 to 6 h	0 to 12 h	0 to 21 h
IS5/IS1031	6.28	1.67	1.01
IS630	5.64	1.68	1.62
IS982	3.50	-0.28	0.51
ISL3	2.57	0.26	2.30

^1^The average fold change in RPKM for all members of each transposon family is shown. Most RNA-seq reads align to multiple members of a highly conserved transposon family; therefore, we cannot report fold change in RPKM for single members of a family. Gene members of the IS5/IS1031 family are all2692, all2693, alr3610, alr3611, all4399, all4400, alr4438, alr4439, all4816, all4817, alr5157, and alr5158. Gene members of the IS630 family are alr0018, alr0019, all0362, all0363, alr0552, alr0553, alr1726, alr1727, alr1853, alr1854, alr1858, alr1859, all1971, all1972, asl1992, all2066, all2067, alr2773, alr2774, alr4628, all4867, all4868, alr5227, and alr5228. Gene members of the IS982 family are asl0588, alr0590, alr0999, all2664, alr2683, alr2694, alr3384, all3624, and alr4082. Gene members of the ISL3 family are alr1609 and alr2698.

### Transcript mapping

**Figure 2:**
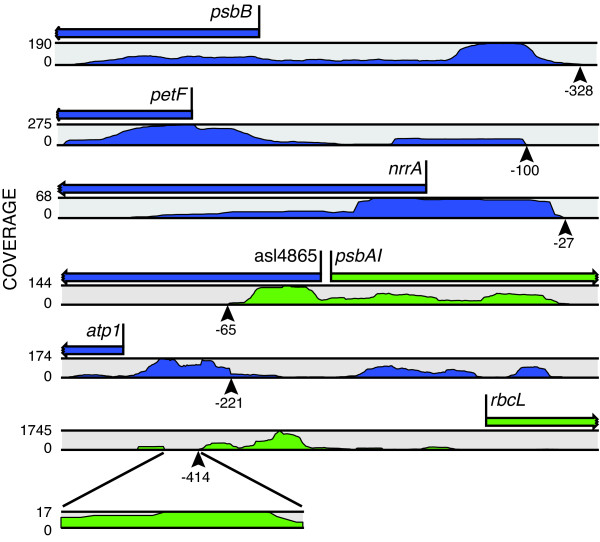
**Transcript 5' ends of *psbB*, *petF*, *nrrA*, *psbAI*, *atp1*, and *rbcL***. RNA-seq coverage at each base along a gene's ORF and UTR are mapped below the gene. RNA-seq reads for *psbB, petF, nrrA*, and *psbAI *show clear 5' ends (characterized by an abrupt drop to 0 coverage) at -328, -100, -27, and -65, respectively, which correspond with published results obtained with other methods. However, *atp1 *and *rbcL *show trailing reads without a complete break upstream of the mapped 5' ends at -221 and -414, respectively, indicating that transcription originates further upstream. RNA abundance along a transcript varies with message stability, often resulting in higher coverage at the 5' end of messages. Scales on left are reads per base position. All data shown are from the 0 h time point with the exception of nrrA, which is from the 21 h time point. Green and blue, ORF orientation and RNA-seq reads to the right and left, respectively.

**Figure 3:**
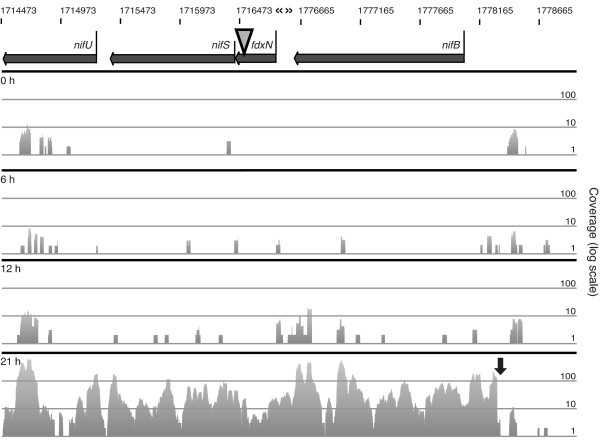
**Analysis of the *nifB-fdxN-nifS-nifU *operon structure, 5' end, and expression levels in response to nitrogen deprivation**. RNA-seq read coverage is shown on a log scale across the *nifB-fdxN-nifS-nifU *operon in heterocyst chromosomes for 0, 6, 12, and 21 hours after the removal of combined nitrogen. The log scale is required to allow depiction of both the low numbers of reads at earlier time points and the large increase in reads at 21 h. The 59,428-bp *fdxN *element (denoted by the gray triangle) is excised from the chromosome in heterocysts [6], and the break in nucleotide numbering for the vegetative-cell chromosome is marked with «». The position of the previously mapped mRNA 5' end for the operon is at 282 bases upstream of the *nifB *start codon (marked by the black downward arrow in the 21 h graph) [30].

Unlike previous whole-transcriptome analyses in *Anabaena*, deep sequencing provides information on all transcripts and can help identify 5' and 3' ends and characterize operon structure; however, transcriptional start sites versus processing sites cannot be differentiated with these methods. We used RNA-seq to identify distinct 5' ends for many transcripts, characterized by a set of reads with a common 5' end and the absence of upstream reads (GEO accession #GSE26633). These 5' ends often corresponded with published transcriptional start and/or processing sites; for example *psbB*, *petF*, *nrrA*, *psbAI*, and *nifB*, all of which were previously analyzed via primer extension [30-35] (Figure 2 and 3).

However, for other genes such as *atp1 *(ATP synthase) and *rbcL *(carbon fixation), the pattern of reads showed evidence for transcripts extending upstream from published 5' ends (Figure 2). For both *atp1 *and *rbcL*, there is a considerable drop in read coverage at the previously identified 5' end, but there is still significant coverage upstream of these sites. This suggests that the previously identified 5' end may be a processing site or a site of RNA secondary structure that affects primer extension results on full-length transcripts, and that some of these transcripts originate at upstream start sites. Our analysis of transcript 5' ends suggests that transcription initiation and transcript processing in *Anabaena *is often complex, and that many transcripts have long 5' UTRs with unclear transcriptional start sites or processing sites. These "trailing" 5' ends make identification of transcriptional start sites via primer extension or RACE difficult for some genes, and RNA-seq will be the method of choice for mapping transcripts.

The RNA-seq data can be used to map transcripts and examine the coordinated expression of genes that form operons. For example, the genes in the *nifB-fdxN-nifS-nifU *operon showed very low numbers of reads at 0, 6, and 12 hours during nitrogen step-down, when proheterocysts have yet to create a microoxic environment for nitrogen fixation (Figure 3). By 21 hours, there was a large increase in the number of reads for all four genes, indicating that a single transcript for all four genes originates from a promoter upstream of *nifB*. The RNA-seq data showed a clear 5' end at position -282 upstream of the *nifB *start codon, which validates previously published results [30]. Further research will be required to fully describe the promoters and operon structure for the entire set of nitrogen fixation genes, which may be unexpectedly complex [36]. Additional RNA-seq data or other types of approaches will be required to clarify transcription in certain regions because of fluctuations in read coverage present in our data set. These fluctuations in reads across ORFs and a general increase in coverage around the 5' ends of ORFs are consistent with other RNA-seq datasets [28,37-39]. It is likely that RNA stability and secondary structure contribute to these fluctuations in coverage.

### Identification of antisense RNAs

The small RNA prep protocol maintains information on the direction of transcription by adding different adaptors to the 3' and 5' ends of each RNA molecule in the sample prior to cDNA synthesis. Therefore, we were able to identify antisense RNAs in ORFs or 5' UTRs of annotated genes and also identify transcripts from unannotated regions of the genome (data available at GEO accession #GSE26633). The antisense transcripts would not have been identified with standard microarray or RNA deep sequencing methods because these methods do not normally distinguish between sense and antisense transcripts.

**Figure 4:**
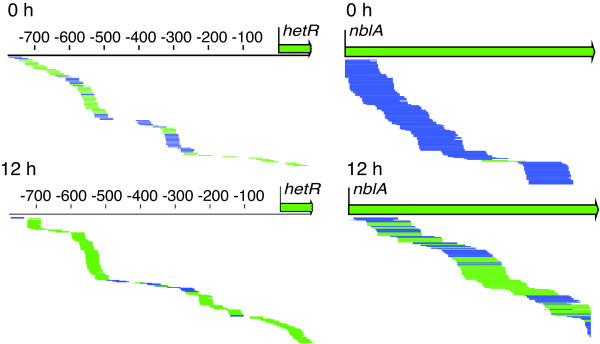
**Examples of antisense RNAs**. RNA-seq reads for 0 and 12 h time points are shown underneath the ORF or 5' UTR as single lines, with blue lines representing reads to the left and green lines representing reads to the right. For the 5' UTR of *hetR *(on left), the ratio of antisense (blue) to sense (green) reads was decreased at 12 h after nitrogen step-down. For *nblA *(on right), antisense reads in the coding region were dominant before nitrogen step-down; however, at 12 h, *nblA *sense reads were abundant and antisense reads were decreased.

Our directional RNA-seq data showed antisense RNAs throughout the *Anabaena *transcriptome (Table 4, Figure 4, and GEO accession #GSE26633). For example, we identified novel antisense transcripts in the 5' ends of key developmental genes such as *hetR *(the master regulator of heterocyst differentiation) and *hetC *(a gene involved in early heterocyst development). Furthermore, we confirmed the presence of the noncoding RNA NsiR1 in the upstream region of *hetF *(another heterocyst regulatory gene) [40-43], and our directional RNA-seq data suggest that the NsiR1 transcript is antisense to the 5' UTR of *hetF*. Other potential noncoding RNAs identified by our directional RNA-seq data include, for example, antisense reads in the region from alr0091 to alr0094 and from alr0709 to alr0710; and abundant rightward reads between alr0249 and all0250, and leftward reads between alr1199 and alr1200 (GEO accession #GSE26633).

**Table 4:** Antisense RNAs transcribed within the ORF or 5' UTR of genes involved in heterocyst differentiation

Gene	Gene Function	Gene Expression After Nitrogen Step-down	RNA Location	Antisense RNA Expression After Nitrogen Step-down
*narB *(alr0612)	nitrate reductase	increased by 12 h	*narB *ORF and 5' UTR	no change
*fraH *(alr1603)	cell-cell connection	increased by 12 h	alr1603 ORF	slight decrease by 21 h
alr3649	heterocyst specific ABC transporter	increased by 12 h	5' end of alr3649 ORF into upstream gene	increased by 12 h
alr3479	similar to nitrogen regulation protein NtrR	decreased slightly at 6 h only	alr3479 ORF	increased by 21 h
*glnB *(all2319)	nitrogen assimilation	no change	*glnB *5' UTR	no change
*hetR *(alr2339)	heterocyst differentiation regulator	increased by 6 h	*hetR *ORF and 5' UTR	decreased by 21 h
*hetC *(alr2817)	heterocyst differentiation	increased by 12 h	two RNAs, one in *hetC *ORF and one in *hetC-hetP *intergenic region	increased by 12 h
*hetF *(alr3546)	heterocyst differentiation	no change	*hetF *ORF and 5' UTR; NsiR1*	increased by 6 h
all3558	nitrogen assimilation regulation	no change	all3558 ORF	increased by 21 h
*hepK *(all4496)	exopolysaccharide synthesis	increased by 12 h	*hepK *ORF; contains repeat sequence at 5' end	no change
*nblA*	phycobilisome degradation	increased by 12 h; largest increase at 21 h	*nblA *ORF and 5' and 3' UTRs	antisense transcription until 12 h only
*hglE *(alr5351)	heterocyst-specific glycolipid	increased by 12 h; largest increase at 21 h	*hglE *3' end of ORF	increased by 12 h
*hglD *(alr5354)	heterocyst-specific glycolipid	increased by 21 h	*hglD *ORF and 5' UTR	no change
*hglC *(alr5355)	heterocyst specific glycolipid gene	increased by 6 h; largest increase at 21 h	*hglC *ORF	increased at 6 h only

*NsiR1 has been previously characterized [40].

Some genes showed striking changes in antisense RNAs. For example, *nblA*, which is involved in degradation of phycobilisome proteins in response to nitrogen deprivation [44], showed extensive antisense reads at 0 hours (Figure 4). After nitrogen step-down, the antisense reads decreased as *nblA *sense reads increased (Figure 4). We hypothesize that it is critical for vegetative cells to avoid expression of even small amounts of NblA protein and that the antisense RNA ensures no expression from the *nblA *gene. The ratio of antisense to sense *nblA *RNA decreases after nitrogen step-down, likely because heterocysts begin to express the coding transcript. Future studies will be required to determine if sense and antisense RNAs for *nblA *are differentially expressed in heterocysts and vegetative cells.

Antisense transcription within genes involved in heterocyst development (Table 4) suggests that antisense RNAs may be an important mechanism of regulation during the response to combined nitrogen deprivation. Future analysis of these noncoding RNAs will shed light on the regulation of genes required for heterocyst development and diazotrophic growth.

## Conclusions

Overall, our data confirm directional RNA deep sequencing as a more thorough method for analyzing transcriptional regulation in cyanobacteria and indicate that further studies using different environmental conditions and mutant strains will yield novel information about *Anabaena *gene regulation. Our RNA-seq data can be used to improve gene annotation and map RNA ends and operon structure. In addition, directional RNA-seq data provide superior information compared to 5' RACE and primer extension experiments for mapping RNA transcripts and identifying potential promoter regions. Because we used a strand-specific sequencing protocol, the dataset can be used to identify antisense and other noncoding RNAs potentially involved in gene regulation. Finally, our work has provided a systems-level view of the *Anabaena *transcriptome during the response to nitrogen step-down. Together, these features of the directional RNA-seq data can be used to define future directions for studying heterocyst development.

## Methods

### Preparation of RNA samples

For deep sequencing, total RNA was prepared from *Anabaena *(*Nostoc*) sp. strain PCC 7120 cultures grown in 100 ml of liquid medium in 250-ml flasks with cotton plugs as previously described with slight modifications [45]. Briefly, 100-ml liquid cultures were grown to an OD_750 _of 0.5 in BG-11(NH_4_) medium, which lacked sodium nitrate and contained 2.5 mM ammonium chloride and 5 mM MOPS (pH 8.0). For the 0 hour sample, cells were collected before deprivation of combined nitrogen. For nitrogen-deprived samples, cells were collected by centrifugation and washed 3 times in BG-11_0_, which lacks sodium nitrate, resuspended in BG-11_0 _to an OD_750 _of approximately 0.05, and incubated for 6, 12, or 21 hours. These times were chosen because at 6 hours cells are beginning to establish a pattern of differentiating cells, at 12 hours proheterocysts are committed to becoming heterocysts [46], and at 21 hours nitrogen-fixing heterocysts are fully differentiated. For each sample, cells were rapidly cooled and collected by pouring 50 ml of culture over 100 g crushed ice and centrifugation at 4,000 × g for 10 minutes at 4°C. Cell pellets were transferred to a 2-ml tube and collected by centrifugation at 11,500 × *g *for 2 minutes at 4°C. Supernatant was removed and cell pellets were flash frozen in liquid nitrogen for storage at -80°C. RNA was isolated from filaments with the Ambion RiboPure RNA isolation kit according to the manufacturer's protocol and total RNA was used for deep sequencing.

### Preparation of a cDNA library

Ribosomal RNA was not removed from total RNA to avoid any depletion of coding transcripts. The presence of ribosomal and transfer RNAs in our sequencing sample did result in a decreased yield of transcript information from coding RNAs (only ~10% of the sample at each time point was non-rRNA and non-tRNA transcripts); however, this protocol avoided the known biases introduced by current rRNA depletion methods [47]. Furthermore, samples were not multiplexed, which allows the sequencing of multiple samples in the same lane, to avoid biases that result from using different adapters for different samples. A directional RNA sequencing library was prepared from 1 μg of total RNA from each sample (0, 6, 12, and 21 h). The RNA samples were purified using a Qiagen RNeasy MinElute Cleanup Kit, fragmented for 180 seconds using a Covaris S2 sonicator set at 10% duty cycle, 5 Intensity, 200 cycles/burst in 120 μl of 1 mM Tris-EDTA pH 8.0 and purified again with the cleanup kit. The fragmented RNA (100 ng) was dephosphorylated using Antarctic phosphatase (2 units, 37°C, 30 min), 5' phosphorylated using T4 polynucleotide kinase (20 units, 37°C, 60 min), and purified with the cleanup kit. cDNA library preparation steps were performed as described in the Illumina Small RNA v1.5 Sample Preparation Guide except that during size selection on 4% agarose gels fragments of approximately 250 bp were obtained, which are suitable for up to 100 base sequencing reads. We saw no evidence of genomic DNA contamination of the RNA samples prior to cDNA synthesis, as there were many regions with no read coverage across the genome.

### Illumina sequencing and analysis

For sequencing, the library was denatured and diluted following standard Illumina-recommended protocols to a final concentration of 9 pM before being loaded onto an Illumina single read flow-cell for massively parallel sequencing on an Illumina GAIIx. Raw sequences were obtained from GA Pipeline software using CASAVA v1.7. The reads were further processed to remove any adapter sequence using the Illumina Flicker add-on. Flicker v2.7 trims the adaptor sequence from each read and does iterative alignment to the reference genome using ELAND.

One sample was sequenced per lane, yielding an average of 17 million high quality 36-bp reads per sample for the 0-, 12-, and 21-hour time points, 1.7 million of which were from non-rRNA and non-tRNA transcripts. The 6-hour sample was sequenced with longer 100-bp reads; however, these reads were trimmed to 40 bp for our analysis. RNA-seq data were aligned and analyzed with CLC Genomics Workbench 4 to create SAM files that were further analyzed with the Cufflinks software suite [27]. CLC genomics workbench 4 was used to generate expression profiles and clustering. Additional files 1, 2, 3, 4, 5, 6, and 7 contain annotated read data, RPKM values, and fold-change calculations for the time-course expression data from the chromosome and six plasmids.

RNA-seq data are available through the NCBI Gene Expression Omnibus (GEO) database, accession number #GSE26633. The raw sequence reads as well BAM files of reads at each time point aligned to NCBI's current build of the *Anabaena *sp. strain PCC 7120 genome are included in the accession. Raw reads are .txt files and can be opened with a FASTA viewer. Aligned reads are in .BAM format and can be analyzed with free or commercial software suites.

## Competing interests

The authors declare that they have no competing interests.

## Authors' contributions

BLF participated in the design of the experiments, collected RNA samples, analyzed sequence data, and drafted the manuscript. FVN constructed the cDNA library. SRH helped design the directional library construction and oversaw Illumina sequencing. JWG participated in design of the experiments and helped draft the manuscript. All authors read and approved the final manuscript.

## Supplementary Material

Additional file 1 **Chromosome RNA-seq data**. Chromosome RNA-seq data for *Anabaena *PCC 7120 at 0, 6, 12, and 21 hours after nitrogen step-down. Click here for file

Additional file 2 **Alpha plasmid RNA-seq data**. Alpha plasmid RNA-seq data for *Anabaena *PCC 7120 at 0, 6, 12, and 21 hours after nitrogen step-down. Click here for file

Additional file 3 **Beta plasmid RNA-seq data**. Beta plasmid RNA-seq data for *Anabaena *PCC 7120 at 0, 6, 12, and 21 hours after nitrogen step-down. Click here for file

Additional file 4 **Gamma plasmid RNA-seq data**. Gamma plasmid RNA-seq data for *Anabaena *PCC 7120 at 0, 6, 12, and 21 hours after nitrogen step-down. Click here for file

Additional file 5 **Delta plasmid RNA-seq data**. Delta plasmid RNA-seq data for *Anabaena *PCC 7120 at 0, 6, 12, and 21 hours after nitrogen step-down. Click here for file

Additional file 6 **Epsilon plasmid RNA-seq data**. Epsilon plasmid RNA-seq data for *Anabaena *PCC 7120 at 0, 6, 12, and 21 hours after nitrogen step-down. Click here for file

Additional file 7 **Zeta plasmid RNA-seq data**. Zeta plasmid RNA-seq data for *Anabaena *PCC 7120 at 0, 6, 12, and 21 hours after nitrogen step-down. Click here for file

Additional file 8 **RPKM heat map data**. Change in RPKM data for *Anabaena *PCC 7120 from 0 to 6, 12, and 21 hours after nitrogen step-down used to prepare heat map. Click here for file
